# Evaluation of Real-World Patient Engagement in a Remote Patient Monitoring for Hypertension Program

**DOI:** 10.1007/s11606-026-10303-y

**Published:** 2026-03-02

**Authors:** Ashley A. Berlot, Allison Stark, Rakin Islam, Chenshu Zhang, Carlos J. Rodriguez, Sharon Rikin

**Affiliations:** 1 Albert Einstein College of Medicine, Bronx, NY USA; 2 Beth Israel Deaconess Medical Center, Boston, MA USA; 3 Montefiore Health System, Bronx, NY USA

**Keywords:** hypertension, equity, engagement, remote patient monitoring, blood pressure measurement

## Abstract

**Background:**

Remote patient monitoring (RPM) for hypertension is effective for lowering blood pressure (BP), but it is unknown whether engagement in RPM is equal for diverse populations or whether engagement impacts achievement of BP targets.

**Objective:**

To determine how participant characteristics are associated with level of RPM engagement and examine whether RPM engagement impacts achievement of BP < 140/90 mmHg at six months.

**Design:**

Retrospective cohort study from January 1, 2022 to August 31, 2023.

**Participants:**

Adults with hypertension, referred to RPM by their primary care clinician with the opportunity to be enrolled for least six months of enrollment at an urban academic health center.

**Main Measures:**

The primary outcome was a high level of engagement, defined as measuring BP more than half the days of the month for at least two of the three first months of participation, versus a low level of engagement. The secondary outcome was BP control < 140/90 mmHg by six months.

**Key Results:**

A total of 835 patients were enrolled in RPM. There were 521 (62.40%) participants with a high level of engagement. Older age (aOR = 1.03, 95% CI [1.01, 1.04]) and lower baseline average systolic BP (aOR = 0.98, 95% CI [0.97, 0.99]) were associated with increased odds of a high level of engagement. Gender, race/ethnicity, and preferred language were not associated with level of engagement. Among all participants, 478 (62.65%) achieved BP control by six months. Those with a high level of engagement, adjusted for age, gender, race/ethnicity, and baseline average systolic BP, had 83% higher odds of achieving BP control compared to those with a low level of engagement (aOR = 1.83, 95% CI [1.24, 2.69]).

**Conclusions:**

RPM engagement was feasible in a diverse patient population and was similar across demographic groups. A higher level of engagement was associated with achieving BP control.

**Supplementary Information:**

The online version contains supplementary material available at 10.1007/s11606-026-10303-y.

## INTRODUCTION

Hypertension impacts approximately 77 million US adults with disparities by race/ethnicity for hypertension prevalence and control^1^. Current guidelines recommend both in-office and home blood pressure (BP) monitoring to manage hypertension^2–5^. One method of home BP monitoring is remote patient monitoring (RPM). RPM is performed with home BP measurements that are directly transmitted to an online portal or electronic medical record for clinical teams to review. RPM can be coupled with rapid follow-up and team-based care for titration of antihypertensive medication, management of elevated BP, and encouragement of lifestyle management^5–9^. However, RPM requires participants to engage in home BP monitoring to accurately obtain measurements, and participants are required to measure their BP 16 or more days a month to support the sustainability of RPM programs through billing and reimbursement^10^. Whether engagement in RPM programs is similar in diverse populations and whether high level of engagement is required to achieve BP improvement is not well established.

Health-related social needs impact BP control, with health disparities often highest among those who identify as Black or Hispanic/Latino^1,11–13^. RPM programs have an opportunity to improve equity for hypertension management by overcoming known barriers that disproportionally impact historically marginalized populations’ ability to engage in traditional office-based hypertension management, including transportation, lack of BP treatment intensification, and challenges with self-management^14–17^. There is conflicting evidence on whether participant populations with various medical and sociodemographic factors engage similarly with RPM programs^6,8,9,18–25^.

As part of an urban academic health system’s initiative to improve BP control among primary care patients, a Montefiore Medical Center RPM for hypertension (RPM-HTN) program was implemented in 2021. The program was designed to address known patient barriers and facilitators for hypertension management which are key determinants of inequities in hypertension management. The first objective of this study was to evaluate if this program was, once implemented, successfully designed to ensure equal engagement and BP outcomes. The second objective was to determine if level of engagement in the RPM program was associated with BP improvement. Evaluating RPM level of engagement in a real-world setting can provide evidence for health systems and policy makers to use when designing RPM programs and establishing billing requirements to meet diverse patient needs and enhance effectiveness of RPM programs.

## METHODS

### Study Design, Setting and Participants

This retrospective cohort study is an analysis of data from participants with the opportunity to be enrolled for six or more months in a primary care RPM program at Montefiore Medical Center between January 1, 2022 and August 31, 2023. The health system has a network of 20 primary care sites serving a predominantly low-income racially and ethnically diverse population often facing social inequities. The patient population has a high prevalence of hypertension, and most patients (> 80%) have government insurance. The study was approved by the Albert Einstein College of Medicine Institutional Review Board.

### RPM for Hypertension Program

RPM-HTN was implemented at five primary care outpatient clinics in 2021^26^. Adult patients with hypertension could be referred to RPM-HTN by their primary care provider (PCP). Exclusions for enrollment in RPM-HTN included end-stage kidney disease and inability to perform self-measured BP. At the time of referral, insurance coverage of RPM services was verified with patients, and patients who agreed to participate were enrolled. During the study period, we received funding from the National Hypertension Control Initiative that was used to cover the cost of RPM services for uninsured patients.

RPM-HTN included 1) participant use of cellular-enabled home BP device, 2) monthly BP measurement review with a team of nurses regardless of number of BP readings, 3) monthly medication management with clinical pharmacists for patients with BP ≥ 140/90 mmHg, and 4) linkage to resources for health-related social needs and health coaching with community health workers. The pharmacists targeted a BP of either < 140/90 or < 130/80 mmHg based on PCP request at the time of ordering RPM-HTN. This study was conducted according to 2017 American College of Cardiology guidelines, prior to the updated U.S. hypertension guidelines recommending < 130/80 for all people with hypertension^27^. All RPM hypertension components were delivered remotely through telephone visits. Participants were instructed according to take at least two readings one or two minutes apart in the morning before taking medications, and in the evening before dinner each day based on the American Heart Association Blood Pressure Measurement Instructions^28^. If they did not measure their BP for more than two days, participants received nudges encouraging engagement through SMS/text messages, e-mails, and/or telephone calls.

### Measures and Data Sources

The primary outcome was level of RPM engagement. The Centers for Medicare and Medicaid Services (CMS) required data transmission over at least 16 days of a 30-day period to qualify for RPM billing^10^. Therefore, level of engagement was defined as high if participants recorded BP data for greater than half the days in a calendar month (month 1: > 50% of days enrolled; month 2 and 3: ≥ 16 days a month) for at least two of the three first months of use. Level of engagement was defined as low if participants did not meet the high level of engagement criteria. In exploratory analyses, level of engagement was defined as high if there were at least seven or two days of BP measurement for two of the first three months of enrollment based on guidelines for self-measured BP monitoring which recommend utilizing the average BP from seven days of readings and updated CMS policies RPM^5^^,28^^,29^.

The secondary outcome of BP improvement was defined in two ways. 1) achievement of BP control < 140/90 mmHg by month six of participation, based on the Healthcare Effectiveness Data and Information Set (HEDIS) BP control value < 140/90 mmHg and 2017 American College of Cardiology consensus guidelines for stage 2 hypertension and 2) change in systolic BP between month one and month six of enrollment^30^.

Participant characteristics of age, gender, race/ethnicity [1) Hispanic, 2) non-Hispanic Black or African American, or 3) other which included non-Hispanic White, Asian, other categories or unknown], preferred language, and hypertension-associated comorbidities of diabetes, cardiovascular disease (CVD), or chronic kidney disease (CKD), were extracted from the electronic medical record. Gender, race/ethnicity, and preferred language were self-identified during patient registration. Race/ethnicity is an important variable, as disparities in BP control have persisted in those who are Hispanic or Black, largely driven by structural racism, socioeconomic barriers, and inequities in access to and delivery of healthcare^1^. Monthly average BP data were extracted from the RPM vendor website. Baseline average BP was the average systolic BP during month one of RPM BP measurement, with baseline systolic BP control dichotomized as ≥ or < 140 mmHg. Baseline systolic BP was kept as a continuous variable for adjustment in models.

### Statistical Analysis

For the primary outcome, bivariate logistic regression was performed to decide which variables to include in the multivariate logistic regression, including variables with significant association and a priori identified variables age, gender, race/ethnicity, preferred language, and baseline starting systolic BP to determine if each participant characteristic was associated with level of engagement. Likelihood ratio (LR) testing determined if there was an interaction with age and race/ethnicity or age and preferred language.

For the secondary outcomes, logistic regression was used to determine whether level of engagement was associated with BP control (< 140/90 mmHg) by six months. Linear regression was used to determine whether level of engagement was associated with change in average systolic BP (continuous variable) by six months. The evaluation of improvement of BP was the difference from baseline to six-month average systolic BP with a minimum of two months of data required to be included in the analysis. If there were no measurements in the sixth month of participation, the most recent prior month with non-missing data was used (e.g., month five). We utilized multivariate logistic regression models to evaluate the relationship between level of engagement and BP control with our primary and exploratory definitions of high engagement. The two-tailed p value for significance was *p* < 0.05 and all analysis was performed on STATA 17SE (College Station, TX).

## RESULTS

There were 991 patients referred during the study period who could have been eligible for six or more months of enrollment; among these 835 agreed to participate and were enrolled in RPM-HTN and were included in this study. Participants were on average 63 years old (SD 14) and mostly female (67.90%). Participants self-identified as Hispanic (43.59%), non-Hispanic Black or African American (40.36%), or in a heterogeneous other category (non-Hispanic White, Asian, other, and unknown) (16.1%) The baseline average systolic BP was 141 mmHg (SD 16) and 56.3% of participants had a baseline average BP ≥ 140/90. Among participants with baseline average BP ≥ 140/90, the average systolic BP was 151 mmHg (SD 12).

### Participant Characteristics and Level of Engagement in RPM-HTN

There were 521 (62.40%) participants with a high level of engagement. The mean (standard deviation) for high engagement each month for the first three months was as follows: 12 days in month one (± 8 days); 25 days in month two (± 5 days); 23 days in month three (± 7 days); for low engagement: 4 days in month one (± 5 days); 7 days in month two (± 7 days); 7 days in month three (± 7 days). The first month of participation was a partial month for the majority of participants. Participant characteristics by level of engagement are shown in Table 1. In multivariate adjusted logistic regression models, older age (aOR = 1.03, 95% CI [1.01, 1.04], p < 0.001) and lower baseline average systolic BP (aOR = 0.98, 95% CI [0.97, 0.99], *p* < 0.001) were independently associated with higher odds of high level of engagement (Table 2). This model shows that for each decade of age increase in a participant, the odds of higher engagement increased by 34%. There was no interaction with age and race/ethnicity (lr test *p *= 0.37) nor age and preferred language (lr test *p* = 0.98).

**Table 1:** Participant Characteristics and Bivariate Analysis of Association Between Patient Characteristics and Level Of Engagement

	**Total** **(N = 835)**	**Low engagement** **(N = 314)**	**High engagement** **(N = 521)**	**High engagement vs. low engagement odds ratio, 95% CI, p value**
**Age (years, mean ± SD)**	63 ± 14	62 ± 15	64 ± 12	1.01 (1.00, 1.02), *p* = 0.03
**Gender, (%)**				
Female	567 (67.90)	213 (67.83)	354 (67.95)	Reference
Male	268 (32.10)	101 (32.17)	167 (32.05)	0.99 (0.74, 1.34), *p* = 0.97
**Race/ethnicity, (%)**				
Hispanic	364 (43.59)	153 (48.73)	211 (40.50)	Reference
Non-Hispanic Black or African American	337 (40.36)	118 (37.58)	219 (42.03)	1.35 (0.99, 1.83), *p* = 0.06
Other	134 (16.05)	43 (13.69)	91 (17.47)	1.53 (1.01, 2.33), *p* = 0.045
**Preferred Language, (%)**				
English	631 (75.57)	229 (72.93)	402 (77.16)	Reference
Not English	204 (24.43)	85 (27.07)	119 (22.84)	0.80 (0.58, 1.10), *p* = 0.17
**Comorbidity, n(%)**^**a**^	584 (69.94)	218 (69.43)	366 (70.25)	1.04 (0.77, 1.41), *p* = 0.80
**Baseline average systolic blood pressure (continuous variable), (mmHg, mean ± SD)**^**b**^	141 ± 16	144 ± 19	139 ± 15	0.98 (0.97, 0.99), *p* < 0.01
**Baseline systolic blood pressure level of control (dichotomized variable), n(%)**^**c**^				
≥ 140/90 mmHg	381 (56.28)	133 (68.21)	248 (51.45)	Reference
< 140/90 mmHg	296 (43.72)	62 (31.79)	234 (48.55)	2.02 (1.43, 2.87), *p *< 0.01

^**a**^Comorbidity included cardiovascular disease, chronic kidney disease, and diabetes and only number and percent of those with a comorbidity is shown

^**b**^Month one baseline systolic blood pressure is defined as average systolic blood pressure in month one of study, regardless of number of days of use, *N* = 678

^**c**^*N* = 677 (one person excluded for “low” average BP classification)

**Table 2:** Association of Participant Characteristics with Engagement for Those with the Opportunity to be Enrolled in Remote Patient Monitoring (RPM) for at Least Six Months

	**High engagement vs. low engagement adjusted odds ratio, 95% CI, p value**
Age (years)	1.03 (1.01, 1.04), *p* < 0.001
Gender	
Female	Reference
Male	1.04 (0.72, 1.50), *p* = 0.82
Race/ethnicity	
Hispanic	Reference
Non-Hispanic Black or African American	1.25 (0.83, 1.88), *p* = 0.28
Other	1.55 (0.93, 2.59), *p* = 0.09
Preferred Language	
English	Reference
Not English	0.80 (0.51, 1.24), *p* = 0.34
Baseline average systolic blood pressure (mmHg)	0.98 (0.97, 0.99), *p* < 0.001

### Level of Engagement and BP Control < 140/90mmHg by Six-months of RPM-HTN Participation

There were 656 participants with at least two months of measurements which were included in the multivariate analyses evaluating if level of engagement is associated with BP control and improvement. There were 489 participants with the last month of data in month six of participation, 112 with the last month of data in month five of participation, 89 with the last month of data in month four of participation, 49 with the last month of data in month three of participation, and 24 with the last month of data in month two of participation. By six months of RPM-HTN participation, 478 (62.65%) of participants achieved BP control < 140/90 mmHg, 355 (68.14%) among those with high level of engagement and 123 (50.83%) among those with low level of engagement. In adjusted models, those with a high level of engagement, had an 83% higher odds of achieving BP control of < 140/90 mmHg compared to those with a low level of engagement (aOR = 1.83, 95% CI [1.24, 2.69], *p* < 0.01) (Table 3). Older age and lower baseline systolic BP were associated with higher odds of achieving BP control of < 140/90 mmHg. In the exploratory analysis with a different threshold of high engagement defined as at least seven days of BP measurement per month instead of 16 days, those with a high level of engagement had 187% higher odds of achieving BP control of < 140/90 mmHg compared to those with a low level of engagement (aOR = 2.87, 95% CI [1.68, 4.91], *p* < 0.01) (Supplemental Table 1). In the exploratory analysis with a different threshold of high engagement defined as at least two days of BP measurement per month (*N* = 710) instead of ≥ 16 days, those with a high level of engagement had a 677% greater odds of achieving BP control of < 140/90 mmHg compared to those with a low level of engagement (aOR = 7.77, 95% CI [2.55, 23.71], *p* < 0.01) (Supplemental Table 2).

**Table 3:** Association of Level of Engagement in Remote Patient Monitoring (RPM) for Hypertension with Blood Pressure < 140/90 mmHg for Those with the Opportunity to be Enrolled in Remote Patient Monitoring (RPM) for at Least Six Months

	**BP < 140/90 mmHg for high vs. low engagement** **OR, 95% CI, p value** **N = 656**
Level of engagement	
Low	Reference
High	1.83 (1.24, 2.69), *p* < 0.01
Age	1.02 (1.01, 1.03), *p* < 0.01
Gender	
Female	Reference
Male	0.80 (0.56, 1.14), *p *= 0.21
Race/ethnicity	
Hispanic	Reference
Non-Hispanic Black or African American	1.25 (0.86, 1.83), *p* = 0.24
Other	1.66 (1.00, 2.75), *p* = 0.05
Baseline SBP	0.95 (0.94, 0.96), *p* < 0.01

### Level of Engagement and Change in BP by Six-months of RPM-HTN Participation

By six months of RPM-HTN participation, average systolic BP change was −7 mmHg among those with high level of engagement and −5 mmHg among those with low level of engagement. Those with a high level of engagement had a greater reduction in mean systolic BP compared to those with a low level of engagement in adjusted models (beta = −4.57, 95% CI [−6.79, −2.34], *p* < 0.01) (Table 4). In the exploratory analysis with a different threshold of high engagement defined as ≥ 7 days of BP measurement per month instead of 16 days, those with a high level of engagement had a greater reduction in systolic BP in adjusted models (beta = −6.18, 95% CI [−9.17, −3.20], *p* < 0.01) (Supplemental Table 2). In the exploratory analysis with a different threshold of high engagement defined as at least two days of BP measurement per month instead of 16 days, those with a high level of engagement had the greatest reduction in mean systolic BP compared to the other definitions of engagement as above in adjusted models (beta = −9.15, 95% CI [−14.39, −3.91], *p* < 0.01) (Supplemental Table 4).

**Table 4:** Association of Level of Engagement in Remote Patient Monitoring (RPM) for Hypertension with Change in Systolic Blood Pressure for Participants with the Opportunity to be Enrolled in Remote Patient Monitoring (RPM) for at Least Six Months

	**Difference in mean SBP for high vs. low engagement** **β, 95% CI, p value** ***N*** = 656
Level of engagement	
Low	Reference
High	−4.57 (−6.79, −2.34), *p* < 0.01
Age	0.01 (−0.07, 0.08), *p* = 0.85
Gender	
Female	Reference
Male	3.54 (1.48, 5.59), *p* < 0.01
Race/ethnicity	
Hispanic	Reference
Non-Hispanic Black or African American	0.01 (−2.11, 2.12), *p* = 0.99
Other	−2.03 (−4.80, 0.74), *p* = 0.15
Baseline SBP	−0.58 (−0.65, −0.52), *p* < 0.01

## DISCUSSION

We found that the majority of RPM participants achieved a high level of engagement with BP measurement. Only older age and lower baseline BP were associated with level of engagement. This is promising for engagement in RPM in other diverse populations and for health systems implementing RPM. We found engagement with BP measurement was associated with higher odds of reaching BP < 140/90 mmHg and greater reduction in systolic BP compared to those with low engagement. In a prior evaluation of RPM-HTN, high engagement with BP measurement and medication titration were the most impactful program components. This suggests patient engagement may facilitate lifestyle changes and medication adherence^31^. Importantly, in the current study, this association persisted when the definition of engagement was lessened from ≥ 16 days of measurement per month as required for billing to at least seven or two days of measurement as recommended by guidelines for self-measured blood pressure and new CMS policies. This suggests that the policy changes allowing for lesser number of days of measurement per month required to bill for RPM services may still be effective for improving BP.

Similar to previous studies, we found older participants were more likely to engage in RPM^25^. Older individuals may have less competing priorities such as work or caregiving that may facilitate engagement^32^. Among patients with lower baseline BP, there may be higher engagement in hypertension management, correlating with engagement in RPM. This study adds to the growing literature demonstrating improvement in BP in real-world RPM programs including urban, rural, predominantly low-income populations^8,23,33–36^.

To our knowledge, there is one similar study investigating the association of level of engagement with improvement of BP at six months of RPM participation^33^. This study found similar levels of engagement among participants (76% averaged ≥ 16 days per month), but no association between level of engagement and improvement in BP^30^. However, this study did not evaluate patient characteristics or the use of RPM in a diverse population^33^. Another study in a health system in an underserved area of a largely low-income and diverse patient population, similar to this study, investigated the feasibility and acceptability of RPM engagement at the threshold of ≥ 16 days per month or a more lenient criteria of at least one day per week for four consecutive weeks^34^. It found only 25.6% met high engagement criteria and 42.4% met more lenient criteria for engagement, suggesting there are barriers to RPM adherence in a low-income, underserved population, though it did not evaluate how specific patient characteristics were associated with engagement^34^. RPM for hypertension programs will likely benefit from responding to real-word evaluations of RPM programs and adapting to participant feedback to increase engagement^19,32^. Importantly, CMS recently published the Medicare Physician Fee Schedule 2026 Proposed Rule for new billing codes for RPM data transmission, allowing Medicare payment if data is collected 2 to 15 days in a 30-day period using a separate CPT code in addition to the current one that requires at least 16 days of data^37^. This additional flexibility for billing RPM services holds promise for adaptable patient engagement models of care.

There are multiple strengths to our study. Our study included longitudinal data over six months. We evaluated multiple benchmarks for engagement, set at 16, seven and two days. However, there are some limitations. There was no control group to compare BP outcomes among those with RPM to those without. Six months is a relatively short-term outcome; further studies are needed to demonstrate feasibility and efficacy with longer-term outcomes. We limited our study to participants who had the opportunity to be enrolled for six months, missing those with very low engagement who were likely disenrolled from the program prior to reaching six months. Analyses of the relationship between engagement and BP improvement required at least two months of RPM measurements, which excludes individuals with even lower engagement. There may also be selection bias in participants who were referred by their PCP and agreed to participate, which may differ from patients not referred or who declined referral^26^.

This study has implications for health systems designing RPM-HTN programs and for payor regulations for RPM engagement. While RPM was feasible in a diverse patient population, programs could design RPM-HTN programs to encourage engagement for younger individuals and those with higher baseline BP. Engagement, even with less days per month than the 16 days required for billing, was associated with RPM effectiveness. Because engagement is directly linked to financial reimbursement of RPM services, it is a key factor in RPM program sustainability. CMS’s proposed 2026 billing changes for RPM data collection based on as little as two days of data transmission offers great promise for patient-centered models of RPM engagement, as health systems may be able to implement and be reimbursed for RPM with a lower burden of days of measurement required by patients for engagement.

## Supplementary Information

Below is the link to the electronic supplementary material.

Supplementary file1 (DOCX 19 KB)
