# Supplementary material for: Evaluation of Real-World Patient Engagement in a Remote Patient Monitoring for Hypertension Program
Source: J Gen Intern Med. 2026 Mar 2;41(7):1892–8. doi: 10.1007/s11606-026-10303-y (PMC13176440; doi:10.1007/s11606-026-10303-y)
Supplement: Supplementary file 1 — Supplementary file1 (DOCX 19 KB) [file 11606_2026_10303_MOESM1_ESM.docx]

**Supplemental Table 1.** Exploratory analysis with high engagement defined as ≥ 7 days of measurement per month. Association of level of engagement in remote patient monitoring (RPM) for hypertension with blood pressure <140/90 mmHg for participants with the opportunity to be enrolled in remote patient monitoring (RPM) for at least six months.

|  | **BP <140/90 mmHg for high vs. low engagement**  **OR (95% CI), p value**  **N=656** |
| --- | --- |
| **Level of engagement** |  |
| **Low** | Reference |
| **High** | 2.87 (1.68, 4.91), p<0.01 |
| **Age** | 1.02 (1.01, 1.04), p<0.01 |
| **Gender** |  |
| **Female** | Reference |
| **Male** | 0.80 (0.56, 1.15), p=0.23 |
| **Race/ethnicity** |  |
| **Hispanic** | Reference |
| **Non-Hispanic Black or African American** | 1.26 (0.86, 1.84), p=0.23 |
| **White/Other** | 1.67 (1.01, 2.78), p=0.05 |
| **Continuous variable: Average Baseline SBP** | 0.95 (0.94, 0.96), p<0.01 |

**Supplemental Table 2.** Exploratory analysis with high engagement defined as ≥2 days of measurement per month. Association of level of engagement in remote patient monitoring (RPM) for hypertension with blood pressure <140/90 mmHg for participants with the opportunity to be enrolled in remote patient monitoring (RPM) for at least six months.

|  | **BP <140/90 mmHg for high vs. low engagement**  **OR (95% CI), p value**  **N=656** |
| --- | --- |
| **Level of engagement** |  |
| **Low** | Reference |
| **High** | 7.77 (2.55, 23.71), p<0.01 |
| **Age** | 1.02 (1.01, 1.04), p<0.01 |
| **Gender** |  |
| **Female** | Reference |
| **Male** | 0.77 (0.54, 1.11), p=0.16 |
| **Race/ethnicity** |  |
| **Hispanic** | Reference |
| **Non-Hispanic Black or African American** | 1.31 (0.90, 1.92), p=0.16 |
| **White/Other** | 1.73 (1.04, 2.87), p=0.03 |
| **Continuous variable: Average Baseline SBP** | 0.95 (0.94, 0.96), p<0.01 |

**Supplemental Table 3.** Exploratory analysis with high engagement defined as ≥ 7 days of measurement per month. Association of level of engagement in remote patient monitoring (RPM) for hypertension with change in systolic blood pressure for participants with the opportunity to be enrolled in remote patient monitoring (RPM) for at least six months.

|  | **Difference in mean SBP for high vs. low engagement**  **β, 95% CI, p value**  **N=656** |
| --- | --- |
| **Level of engagement** |  |
| **Low** | Reference |
|  | **Difference in mean SBP for high vs. low engagement**  **β, 95% CI, p value**  **N=656**  **(continued)** |
| **High** | -6.18 (-9.17, -3.20), p<0.01 |
| **Age** | -0.01 (-0.08, 0.07), p=0.81 |
| **Gender** |  |
| **Female** | Reference |
| **Male** | 3.51 (1.46, 5.56), p<0.01 |
| **Race/ethnicity** |  |
| **Hispanic** | Reference |
| **Non-Hispanic Black or African**  **American** | -0.05 (-2.16, 2.06), p=0.96 |
| **White/Other** | -2.05 (-4.81, 0.72), p=0.15 |
| **Continuous variable: Average Baseline SBP** | -0.58 (-0.65, -0.52), p<0.01 |

**Supplemental Table 4.** Exploratory analysis with high engagement defined as ≥2 days of measurement per month. Association of level of engagement in remote patient monitoring (RPM) for hypertension with change in systolic blood pressure for participants with the opportunity to be enrolled in remote patient monitoring (RPM) for at least six months.

|  | **Difference in mean SBP for high vs. low engagement**  **β, 95% CI, p value**  **N=656** |
| --- | --- |
| **Level of engagement** |  |
| **Low** | Reference |
| **High** | -9.15 (-14.39, -3.91), p<0.01 |
| **Age** | -0.02 (-0.10, 0.05), p=0.55 |
| **Gender** |  |
| **Female** | Reference |
| **Male** | 3.67 (1.61, 5.73), p<0.01 |
| **Race/ethnicity** |  |
| **Hispanic** | Reference |
| **Non-Hispanic Black or African**  **American** | -0.29 (-2.41, 1.83) p=0.79 |
| **White/Other** | -2.32 (-5.09, 0.46), p=0.10 |
| **Continuous variable: Average Baseline SBP** | -0.57 (-0.63, -0.51), p<0.01 |
